# Interaction between Retinoid Acid Receptor-Related Orphan Receptor Alpha (RORA) and Neuropeptide S Receptor 1 (NPSR1) in Asthma

**DOI:** 10.1371/journal.pone.0060111

**Published:** 2013-04-02

**Authors:** Nathalie Acevedo, Annika Sääf, Cilla Söderhäll, Erik Melén, Jami Mandelin, Christina Orsmark Pietras, Sini Ezer, Piia Karisola, Johanna Vendelin, Gustav Boije af Gennäs, Jari Yli-Kauhaluoma, Harri Alenius, Erika von Mutius, Gert Doekes, Charlotte Braun-Fahrländer, Josef Riedler, Marianne van Hage, Mauro D’Amato, Annika Scheynius, Göran Pershagen, Juha Kere, Ville Pulkkinen

**Affiliations:** 1 Translational Immunology Unit, Department of Medicine Solna, Karolinska Institutet, Stockholm, Sweden; 2 Institute of Environmental Medicine, Karolinska Institutet, Stockholm, Sweden; 3 Department of Biosciences and Nutrition, Novum, Karolinska Institutet, Huddinge, Sweden; 4 Sachs’ Children’s Hospital, Stockholm, Sweden; 5 Department of Medicine, Institute of Clinical Medicine, University of Helsinki, Helsinki, Finland; 6 Research Programs Unit, Program for Molecular Medicine, University of Helsinki, and Folkhälsan Institute of Genetics, Helsinki, Finland; 7 Unit of Immunotoxicology, Finnish Institute of Occupational Health, Helsinki, Finland; 8 Division of Pharmaceutical Chemistry, Faculty of Pharmacy, University of Helsinki, Helsinki, Finland; 9 University Children’s Hospital Munich, Munich, Germany; 10 The Institute for Risk Assessment Sciences, Utrecht University, Utrecht, The Netherlands; 11 Swiss Tropical and Public Health Institute, and University of Basel, Basel, Switzerland; 12 Children’s Hospital, Schwarzach, Austria; 13 Clinical Immunology and Allergy Unit, Department of Medicine Solna, Karolinska Institutet and University Hospital, Stockholm, Sweden; Sudbury Regional Hospital, Canada

## Abstract

*Retinoid acid receptor-related Orphan Receptor Alpha* (*RORA*) was recently identified as a susceptibility gene for asthma in a genome-wide association study. To investigate the impact of *RORA* on asthma susceptibility, we performed a genetic association study between *RORA* single nucleotide polymorphisms (SNPs) in the vicinity of the asthma-associated SNP (rs11071559) and asthma-related traits. Because the regulatory region of a previously implicated asthma susceptibility gene, *Neuropeptide S receptor 1* (*NPSR1*), has predicted elements for RORA binding, we hypothesized that *RORA* may interact biologically and genetically with *NPSR1*. 37 *RORA* SNPs and eight *NPSR1* SNPs were genotyped in the Swedish birth cohort BAMSE (2033 children) and the European cross-sectional PARSIFAL study (1120 children). Seven *RORA* SNPs confined into a 49 kb region were significantly associated with physician-diagnosed childhood asthma. The most significant association with rs7164773 (T/C) was driven by the CC genotype in asthma cases (OR = 2.0, 95%CI 1.36–2.93, p = 0.0003 in BAMSE; and 1.61, 1.18–2.19, p = 0.002 in the combined BAMSE-PARSIFAL datasets, respectively), and strikingly, the risk effect was dependent on the Gln344Arg mutation in *NPSR1*. In cell models, stimulation of NPSR1 activated a pathway including *RORA* and other circadian clock genes. Over-expression of RORA decreased *NPSR1* promoter activity further suggesting a regulatory loop between these genes. In addition, *Rora* mRNA expression was lower in the lung tissue of *Npsr1* deficient mice compared to wildtype littermates during the early hours of the light period. We conclude that *RORA* SNPs are associated with childhood asthma and show epistasis with *NPSR1,* and the interaction between RORA and NPSR1 may be of biological relevance. Combinations of common susceptibility alleles and less common functional polymorphisms may modify the joint risk effects on asthma susceptibility.

## Introduction

The Retinoic acid receptor-related Orphan Receptor Alpha (*RORA;* MIM 600825, chromosome 15q22.2) was recently implicated in asthma susceptibility by a genome-wide association study (GWAS) [1]. In humans, *RORA* encodes four isoforms (RORA-1, RORA-2, RORA-3, and RORA-4) [2], and the asthma-associated single nucleotide polymorphism (SNP) rs11071559 is located in the first intron of the transcript variant encoding RORA-1. RORA is a transcription factor that belongs to the nuclear hormone-receptor superfamily (NR1) and binds as monomers to specific hormone response elements (RORE) in DNA. By interacting with co-activators and co-repressors [3], RORA might enhance or repress the transcription of target genes [4]–[10]. RORA is mostly known for its functions as a regulator of circadian rhythms, metabolism, as well as mood disorders, and its role in the immune system and asthma has gained attention only recently [3], [11]. *RORA* mRNA expression is positively correlated with gestational age and is differentially expressed during human lung development [12]. The mice deficient of *Rora* (“Staggerer”, *Rora* ^sg/sg^) showed attenuated pulmonary infiltration, less mucous hyperplasia and reduced levels of T helper type 2 (Th2) cytokines interleukin 4 (IL-4), IL-5 and IL-13 in a model of ovalbumin-induced airway inflammation [13]. In addition, RORA participates in the lineage commitment of Th17 cells [14], [15] and is required for the development of nuocytes [16] which are lymphoid-derived innate cells that contribute to the development of asthmatic responses in mouse models of asthma [17]. Nuocytes are activated by binding of IL-33 to its receptor ST2 (IL*-*1RL1) and these cells are a major source of IL-5 and IL-13 in the lung [18]. Thus, the asthma loci *IL33*, *IL1RL1*/*IL18R1*, *RORA*, and *IL13* previously identified by GWAS belong to the same pathway, and could modify airway inflammation and interleukin responses that are crucial for the development of asthma [1], [19]. A meta-analysis of GWAS data has confirmed the putative role of common genetic variation within *RORA* in asthma susceptibility of European Americans [20]. Moreover, a recent GWAS for lung function identified a SNP (rs1902618) within the intron 1 of *RORA* as a possible predictor of age-related decrease in forced expiratory volume in 1 second (FEV_1_) [21].

Common genetic variations as analyzed in the GWAS do not explain a substantial proportion of the missing heritability and the disease risk, and therefore epistasis has been proposed as a possible factor [22]. In this framework, several susceptibility alleles of low-to-moderate-effects may modify the disease risk, especially when the candidate genes belong to common biological pathways [23], [24]. We propose that RORA may interact with the G protein-coupled Neuropeptide S Receptor 1 (NPSR1) pathway. NPSR1 and its ligand Neuropeptide S (NPS) are mostly expressed in specific brain regions and affect multiple neuroendocrine and behavioral responses [25]–[29]. *NPSR1* was identified as a susceptibility gene for asthma and related traits by positional cloning and has been replicated in several independent association studies [30]–[36], and marginally supported by GWAS [1]. In addition, *NPSR1* SNPs have shown genetic associations with other inflammatory phenotypes such as inflammatory bowel disease [37], and rheumatoid arthritis [38], [39]. Surprisingly, the functional *NPSR1* SNP rs324981, encoding a substitution of Asn(107)Ile in the putative ligand-binding pocket of NPSR1, was associated with bedtime and sleepiness in a GWAS for sleep and circadian phenotypes [40]. As phenotypical analysis of *Npsr1* knockout mice have thereafter revealed deficits in circadian locomotor activity [41], [42], we hypothesized that the NPS/NPSR1 pathway might regulate *RORA* and other circadian clock genes. Expression of the circadian clock genes oscillates in the hypothalamus by positive and negative transcription/translation feedback loops to govern the endogenous rhythms as well as the extent of inflammation and cytokine balance through hormonal and neuronal connections [43]–[45]. Taken together, both RORA and the NPS/NPSR1 pathway appear to play a part in complex immune-related, behavioral and circadian outcomes, which could all be controlled by the circadian clock. Thus, the aims of this study were: 1) To clarify the potential functional crosstalk between NPSR1 and RORA; 2) To identify the impact of putative *RORA* risk alleles on the susceptibility to asthma and allergic traits; and 3) To evaluate potential gene-gene interaction (epistasis) between *RORA* and *NPSR1* polymorphisms as risk factors for asthma.

## Results

### Stimulation of NPSR1 with NPS Regulates mRNA Expression of RORA and Other Circadian Clock Genes

**Figure 1:**
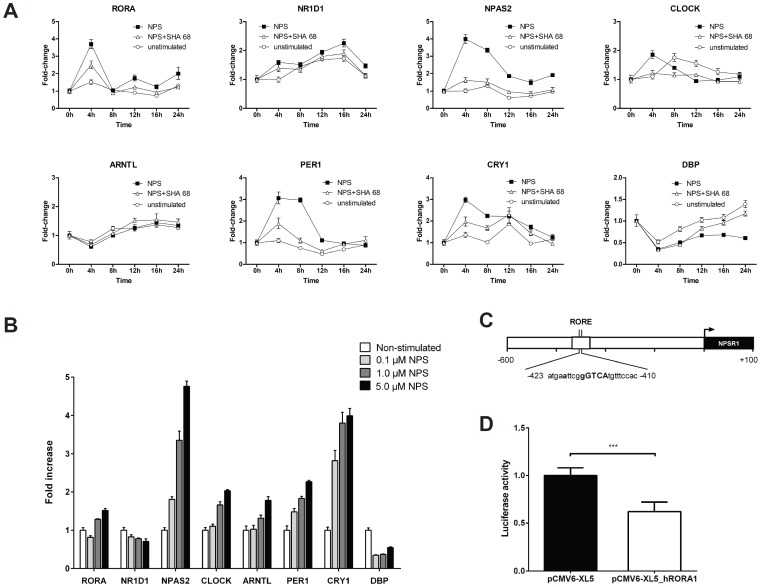
Cross-talk of RORA and NPSR1 in cell models. (**A**) Real-time PCR analysis of circadian clock genes in human SH-SY5Y neuroblastoma cell line over-expressing NPSR1 stimulated with 100 nM NPS for 0–24 h in the presence or absence of 3 µM SHA 68 (*N*-[(4-fluorophenyl)methyl]tetrahydro-3-oxo-1,1-diphenyl-3*H*-oxazolo[3,4-*a*]pyrazine-7(1*H*)-carboxamide), a selective antagonist of NPSR1. (**B**) Real-time PCR analysis of *RORA*, *NR1D1*, *NPAS2*, *CLOCK*, *ARNTL*, *PER1*, *CRY1*, and *DBP* mRNA expression in human embryonic kidney epithelial HEK-293H cell line over-expressing NPSR1 6 h after NPS (0.1–5 µM) stimulation. The results are presented as fold-changes in comparison to the unstimulated cells. GAPDH was used as the endogenous reference, and data are expressed as mean of triplicate samples ±95% confidence intervals. In all experiments, results were calculated with the comparative ΔΔ*C_t_* method. (**C**) Schema of the *NPSR1* promoter and the location of the putative 6-bp AT-rich sequence preceding the half-core motif PuGGTCA (RORE). (**D**) NPSR1 driven luciferase in relative luminescence units after transfection with either RORA-1 encoding plasmid or an empty vector. Ten biological replicates per group. *Mann-Whitney* U test ***p<0.0001.

Human SH-SY5Y neuroblastoma cells over-expressing NPSR1 were stimulated with 100 nM NPS for 0–24 h and the mRNA expression of *RORA* was measured using real-time PCR **(** **Figure 1a** **)**. NPS stimulation increased the expression of *RORA* in SH-SY5Y cells over-expressing NPSR1 in a time and dose dependent manner, and the effect could be partly inhibited by the selective antagonist of NPSR1, SHA 68 (*N*-[(4-fluorophenyl)methyl]tetrahydro-3-oxo-1,1-diphenyl-3*H*-oxazolo[3,4-*a*]pyrazine-7(1*H*)-carboxamide) **(** **Figure 1a** **and File S1)** [46]. These effects were not detected in the parental cell line with very low endogenous NPSR1 expression (data not shown). One of the well-known functions of RORA is the regulation of the circadian clock machinery [43], where it competes with its binding partner NR1D1 (nuclear receptor subfamily 1, group D, member 1) for initiation/inhibition of ARNTL (aryl hydrocarbon receptor nuclear translocator-like) transcription. Since NPS stimulation up-regulated *RORA* expression, we measured the effects of NPS on other components of the clock machinery including *NPAS2* (Neuronal PAS domain-containing protein 2), *PER1* (period 1), *CRY1* (cryptochrome 1) and *DBP* (D site albumin promoter binding protein). NPAS2 can replace CLOCK (circadian locomoter output cycles kaput), and PER1 and CRY1 are regulated by dimerization of the core clock components ARNTL (also known as BMAL1) and CLOCK/NPAS2. As shown in **Figure 1a**, the expression of core clock components *NPAS2*, *PER1*, and *CRY1* was clearly increased after NPS stimulation. In line with this, the expression of *DBP*, an important output gene of the clock, was down-regulated after NPS challenge. The effects of NPS on *NR1D1*, *CLOCK* and *ARNTL* expression were less apparent. The results obtained in the human embryonic kidney epithelial cell line (HEK293H cells) stably over-expressing NPSR1-A confirmed the dose-dependent regulation of *RORA* mRNA and related circadian clock genes 6 h after NPS stimulation **(** **Figure 1b** **).** These results indicated that *RORA* is regulated by the NPS/NPSR1 signaling pathway and suggested a new regulator of circadian clock gene expression.

### RORA Regulates NPSR1 Promoter Activity

The *NPSR1* promoter is predicted to have a RORE binding site **(** **Figure 1c** **)** in the minus strand with a matrix similarity of 0.92 out of 1.0 for RORA and it is found by at least three different prediction methods (MatInspector, Consite, and PScan). To study the effects of RORA on *NPSR1* promoter activity, HeLa cells were co-transfected with (1) the *NPSR1* promoter coupled to a firefly luciferase reporter gene, (2) the plasmid encoding the Renilla luciferase, and (3) either the plasmid encoding the cDNA for RORA-1 (pCMV6-XL5-RORA1) or the plasmid without RORA-1 (pCMV6-XL5) used as a control. As shown in **Figure 1d**, over-expression of RORA-1 led to a significant decrease (37.8±10%; mean±SD) in the luciferase levels compared to the cells co-transfected with the vector without RORA-1. This suggests that RORA-1 binds to the *NPSR1* promoter and down-regulates its activity. This could establish a negative regulatory loop cycling with the circadian oscillation.

### Rora mRNA Expression is Altered in the Lung Tissue of Npsr1 Deficient Mice

**Figure 2:**
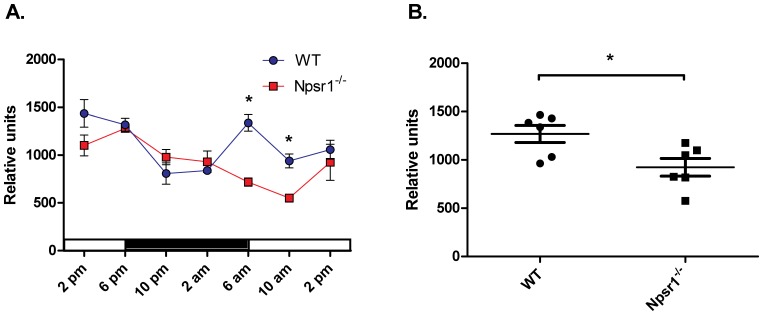
Expression of *Rora* in the lung tissue of wildtype (WT) and *Npsr1* deficient (*Npsr1* ^−/−^) mice. (**A**) Lung tissue was collected from WT and *Npsr1* ^−/−^ mice at 4 h intervals, and diurnal mRNA expression of *Rora* was measured with real-time PCR (n = 3–4/group/timepoint). The black bar represents the dark period. (**B**) Expression of *Rora* in the lung tissue of wildtype and *Npsr1* ^−/−^ mice during the light period (8–12 am) (n = 6). Data are expressed as relative units indicating a fold change in *Rora* mRNA expression that is normalized to an endogenous reference gene (18S ribosomal RNA) and is relative to the non-template control calibrator (mean ± SEM). Similar data were obtained in two separate experiments. *p<0.05.

To address the question whether *Npsr1* knockdown has effects on *Rora* expression *in vivo*, we collected lung tissue at 4 h intervals from *Npsr1* ^−/−^ and wildtype mice during a 24 h period, and measured *Rora* mRNA expression with real-time PCR. As shown in **Figure 2A**, expression of *Rora* is different between wildtype and *Npsr1* deficient mice (n = 3–4/group/timepoint). Because *Rora* mRNA expression was significantly lower in *Npsr1* ^−/−^ mice in comparison to wildtype littermates at the beginning of the light period (subjective night) **(** **Figure 2A** **)**, we verified the results in a separated experiment using a larger group of mice (n = 6/group). Lung samples were collected during the early hours (8–12 am) of the light period. The results confirmed the lower expression of *Rora* in the lung tissue of the *Npsr1* ^−/−^ mice (**Figure 2B**) and correlated with the observed diurnal expression of *Rora*.

### Polymorphisms in RORA are Associated with Asthma but not with Atopic Sensitization

**Figure 3:**
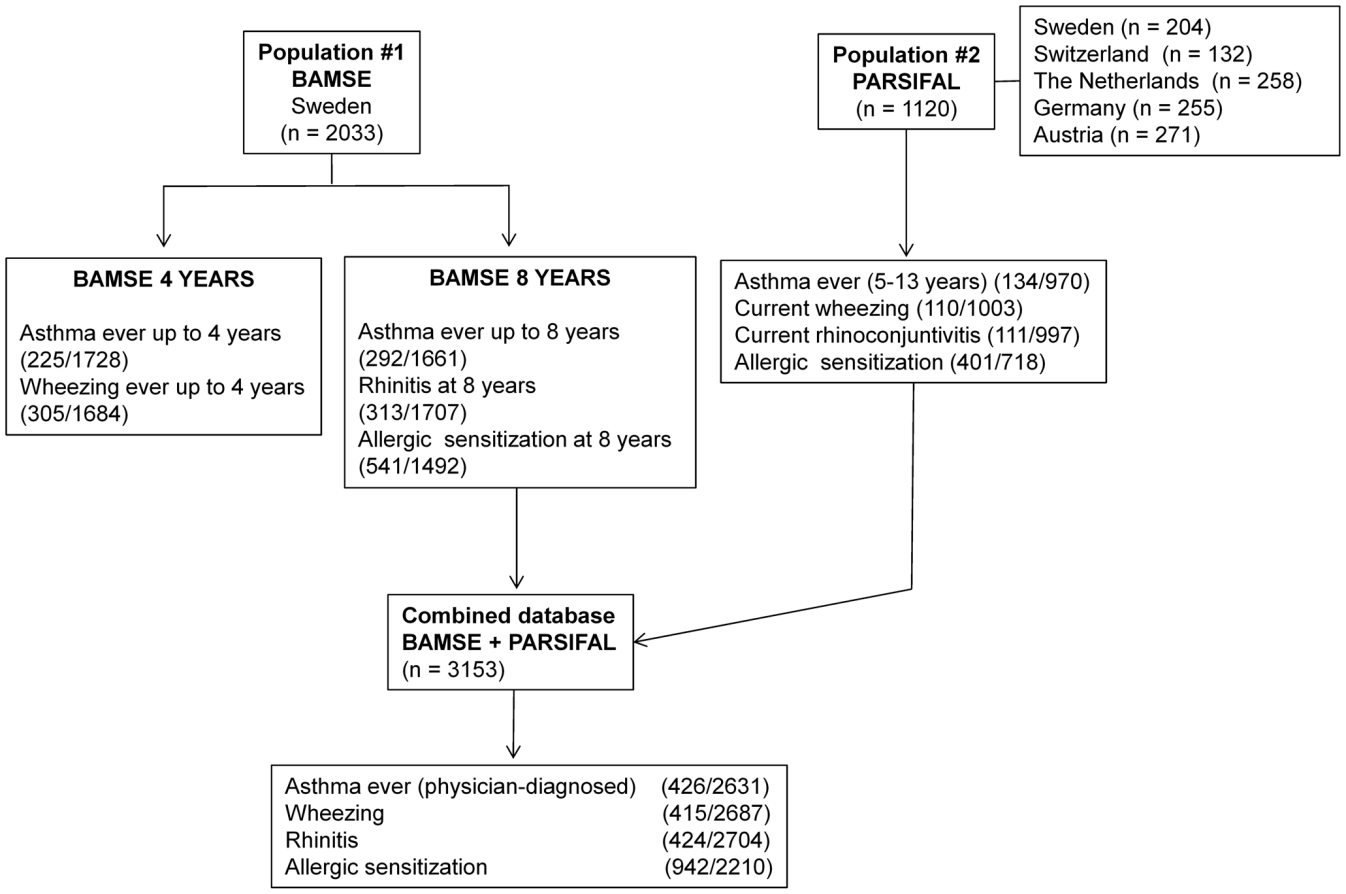
Flow chart with the distribution of cases and controls in the studied populations: BAMSE, PARSIFAL and the combined dataset. Numbers within parenthesis indicate number of children, either as a total or by the presence of a given phenotype (cases/controls).

**Figure 4:**
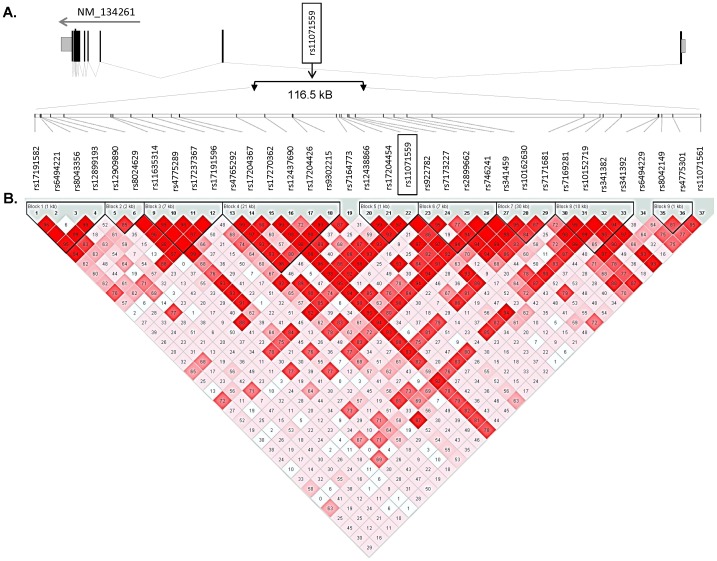
Overview of the genomic location and correlations of the *RORA* SNPs analyzed in this study. (**A**) Schematic representation of the exon intron distribution of the longest isoform of *RORA* (NM_134231) located in chromosome 15q22.2 and the position of the GWAS SNP rs11071559. An expansion of the 116.5 kb region surrounding the rs11071559 reveals the 35 SNPs analyzed in this study according to their positions along the intron 1. (**B**) The LD structure of the 116.5 kb region of RORA as defined by the solid spine algorithm in the combined dataset (n = 3153). Numbers in each box correspond to the pair-wise linkage disequilibrium coefficients (D’) between the respective SNPs. A similar LD structure was observed in BAMSE and PARSIFAL as separated populations. Additional information on the 35 *RORA* SNPs is presented in **Table S3**.

A total of 35 SNPs in *RORA* were analyzed for associations with asthma and allergy-related traits in the prospective Swedish birth-cohort BAMSE (n = 2033) and a subset of the European multicenter cross-sectional study PARSIFAL (n = 1120), **(** **Figure 3** **)**. The SNPs spanned a region of 116.5 kb on chromosome 15q22.2 and tagged the genetic variation in linkage disequilibrium (LD) with the previously associated GWAS SNP rs11071559 **(** **Figure 4** **)**. We first investigated the association between SNPs in *RORA* and physician-diagnosed childhood asthma and other allergic traits in BAMSE and PARSIFAL as independent datasets. The prevalence of the 35 *RORA* SNPs in cases and controls, and the results of the allelic association tests with asthma, wheezing, rhinitis and atopic sensitization are presented in **File S2**.

**Figure 5:**
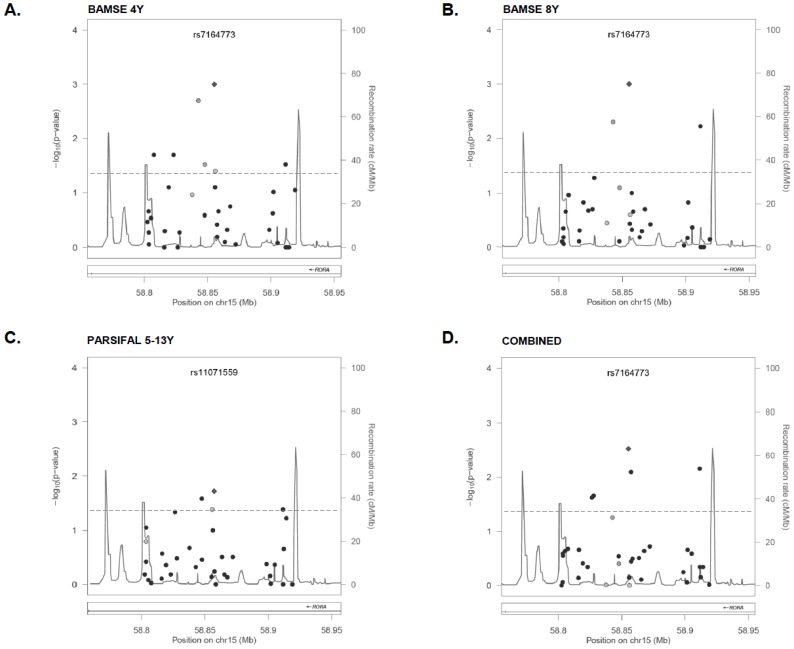
Allelic association tests between *RORA* SNPs and asthma. SNPs are represented by dots in relation to their genomic position. Grey-colored dots have a degree of linkage disequilibrium (r^2^) between 0.40 and 0.60 with the lead SNP (diamond). Recombination rate is indicated as a continuous line. Dashed line represents the significance threshold. (**A**) Asthma ever up to 4 years in BAMSE; (**B**) Asthma ever up to 8 years in BAMSE; (**C**) Physician-diagnosed asthma ever between 5–13 years in PARSIFAL; (**D**) Physician-diagnosed asthma in the combined dataset.

**Figure 6:**
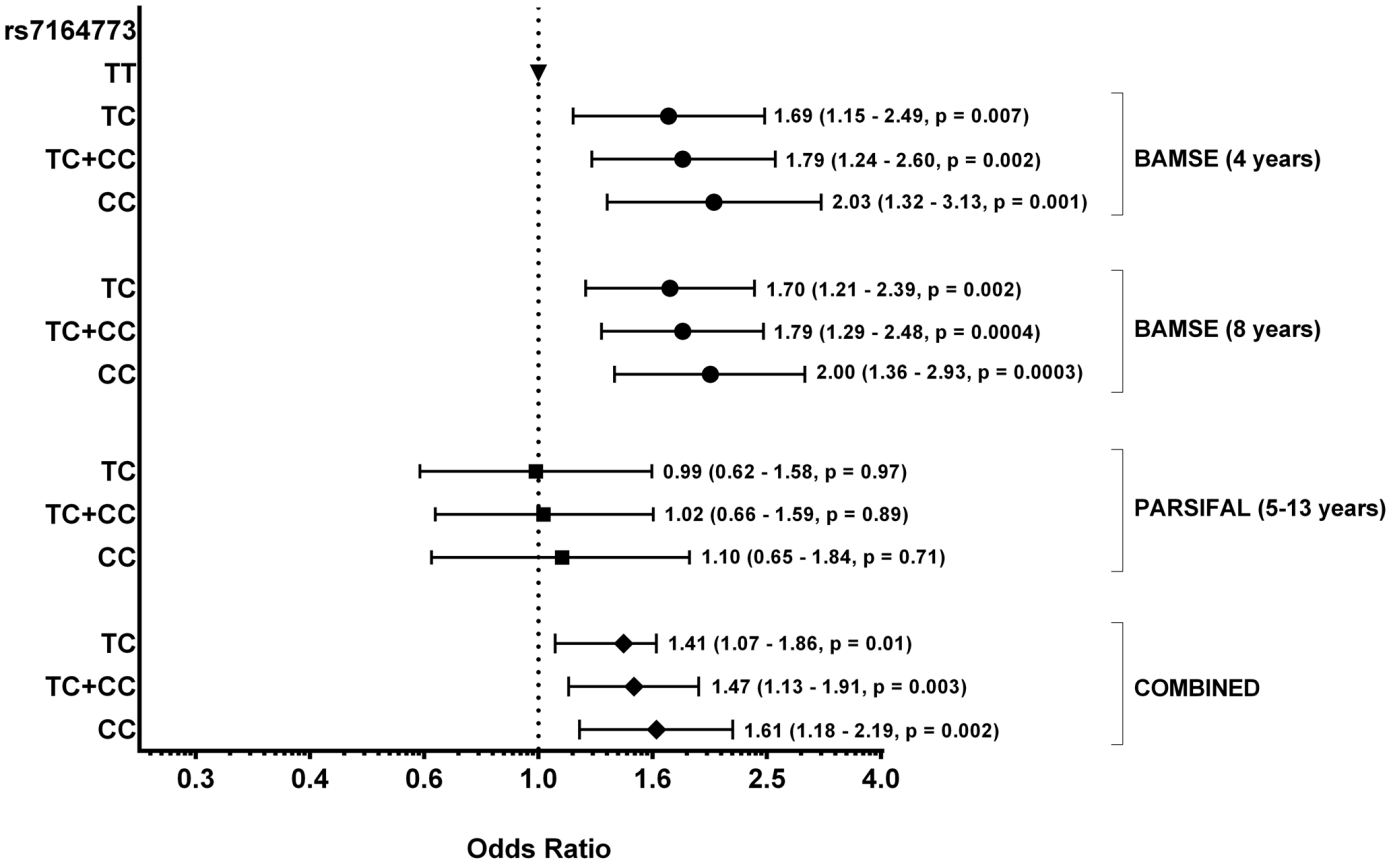
Genotype associations between *RORA* SNP rs7164773 and physician-diagnosed asthma.

In BAMSE, seven *RORA* polymorphisms were associated with the diagnosis of asthma up to 4 years (rs7164773, rs8024629, rs17191596, rs12437690, rs9302215, rs17204454 and rs341392) **(** **Figure 5a** **)**. Three of these SNPs (rs7164773, rs12437690, rs341392) remained associated with the diagnosis of asthma at 8 years **(** **Figure 5b** **)**. Of these, the SNP rs7164773 previously not associated with asthma gave the most significant association signal in BAMSE. There were some associations between *RORA* SNPs and atopic sensitization at 8 years, rhinitis at 8 years and wheezing up to 4 years, but all of them were of borderline significance, suggesting that in this population *RORA* is mainly associated with the diagnosis of childhood asthma rather than atopic-traits **(File S2)**. Similar results were found in PARSIFAL, where four *RORA* SNPs were associated with physician-diagnosed asthma between 5 and 13 years of age (rs11071559, rs4775292, rs17204426 and rs12438866) **(** **Figure 5c** **)**, but there were no associations between *RORA* SNPs and wheezing, rhinoconjuntivitis or atopic sensitization. The GWAS SNP rs11071559 (C>T) was the lead SNP for association with asthma in PARSIFAL as a separate dataset. In the combined BAMSE-PARSIFAL dataset, five *RORA* SNPs including the GWAS rs11071559 were associated with physician-diagnosed asthma and remained significant after adjustment by country of origin **(** **Figure 5d** **)**. Interestingly, none of the *RORA* SNPs were significantly associated with wheezing, rhinitis or atopic sensitization in the combined dataset **(File S2)**. The asthma-associated SNPs, albeit different between BAMSE and PARSIFAL, were confined into a region of 49 kb in the intron 1 of *RORA* (chr15∶61020254–61069988) and their effects were mainly detected under dominant and additive models **(File S3)**. A summary of the most significant *RORA* SNPs associated with asthma is presented in **Table 1**. The *RORA* rs7164773 was the lead SNP in the combined dataset and carriers of one (TC) or two copies (CC) of the rare allele had an increased risk for a physician-diagnosis of asthma **(** **Figure 6** **)**. The association between the lead SNP rs7164773 and asthma was significant after adjustment for the other asthma-associated SNPs *RORA* rs11071559 and *RORA* rs341392. The *RORA* rs11071559 remained marginally associated after adjustment by the lead SNP rs7164773. On the other hand, the association of the *RORA* rs341392 disappeared when adjusting for the lead SNP rs7164773 and for the *RORA* rs11071559. Further information on the genotype distribution in asthmatic cases and controls is presented in **File S4**.

**Table 1:** The *RORA* SNPs associated with asthma and their effects under additive and dominant models.

			Dominant		Additive*				
dbSNP	Genotypes†	Associated phenotype	OR (95%CI),	P value	P value	Dataset	SNP effect	Distance in kb**	EMD prime**
rs8024629	CC/CA/**AA**	Asthma up to 4 years	1.54 (1.15–2.53)	0.003	0.02	B	Increase risk	47.6	0.88
		Asthma up to 8 years	1.34 (1.03–1.75)	0.02	ns	B			
rs17191596	TT/TC/**CC**	Asthma up to 4 years	1.57 (1.16–2.13)	0.003	0.02	B	Increase risk	32.1	0.92
		Asthma up to 8 years	1.29 (0.97–1.71)	0.07	ns	B			
rs12437690	GG/GA/**AA**	Asthma up to 4 years	1.58 (1.16–2.17)	0.004	0.001	B	Increase risk	12.5	0.89
		Asthma up to 8 years	1.42 (1.08–1.87)	0.01	0.004	B			
rs7164773	TT/TC/**CC**	Asthma up to 4 years	1.79 (1.24–2.60)	0.002	0.001	B	Increase risk	–	–
		Asthma up to 8 years	1.79 (1.29–2.48)	0.0004	0.0004	B			
		Asthma ever	1.47 (1.13–1.91)	0.003	0.004	C			
rs12438866	TT/TC/**CC**	Asthma 5–13 years	0.63 (0.43–0.91)	0.01	0.04	P	Protective	0.57	0.31
rs11071559	CC/CT/**TT**	Asthma 5–13 years	0.53 (0.32–0.87)	0.01	0.01	P	Protective	2.04	0.92
		Asthma ever	0.71 (0.55–0.92)	0.01	0.007	C			
rs341392	AA/AC/**CC**	Asthma up to 4 years	0.69 (0.51–0.92)	0.01	0.02	B	Protective	56.2	0.30
		Asthma up to 8 years	0.69 (0.53–0.89)	0.005	0.005	B			
		Asthma ever	0.71 (0.58–0.89)	0.004	0.01	C			

* Exact Armitage P value.

B: Bamse; P: Parsifal, C: combined;

† Rare homozygotes are in bold.

EM: Expectation-Maximization **From the lead SNP rs7164773.

Further information on the genotype distributions is presented in File S3 and File S4.

ns: non-significant.

### Epistasis between RORA and NPSR1 Modify the Risk of Asthma

**Figure 7:**
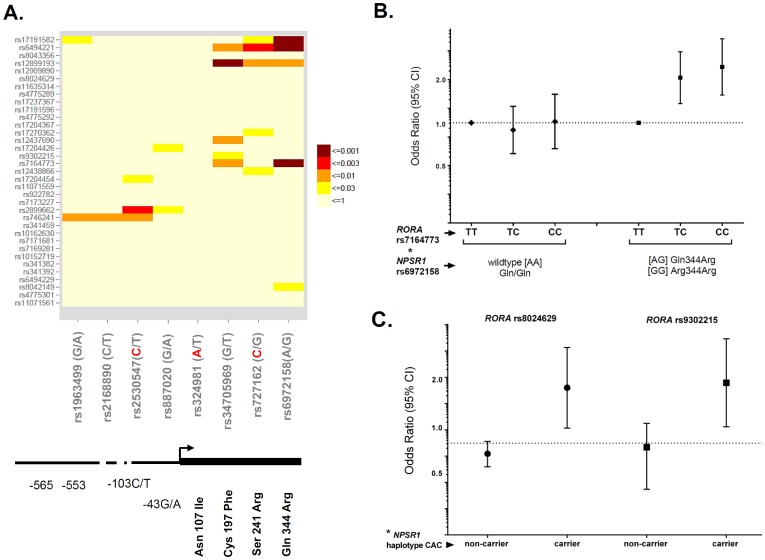
Gene-gene interactions between *RORA* and *NPSR1.* (A) Heatmap of the p-values for interaction between 35 *RORA* SNPs (*y*-axis) and 8 *NPSR1* SNPs (*x*-axis). A schema of the *NPSR1* gene is presented at the bottom of the figure with the relative positions of the SNPs. The alleles comprising the CAC haplotype are marked in red. (B) Effects of one (TC) or two copies (CC) of the *RORA* SNP rs7164773 on asthma risk according to the genotypes on the *NPSR1* SNP rs6972158 (Gln344Arg). Bars represent 95% confidence intervals. (C) Effects of *RORA* rs8024629 and *RORA* rs9302215 on asthma risk according to the functional *NPSR1* haplotype CAC (rs2530547**C**/rs324981**A**/rs727162**C**). All the results for gene-gene interactions analysis are presented after adjustment by age and country of origin.

Based on the results indicating up-regulation of *RORA* mRNA expression after NPS stimulation of NPSR1 over-expressing cells, and the observation that *NPSR1* is down-regulated by RORA, we studied the effects of gene-gene interaction between *RORA* and *NPSR1* SNPs on the risk of asthma. Eight *NPSR1* SNPs [47] spanning a region of 191 kb on chromosome 7p14.3, and including the predicted *NPSR1* promoter and the coding region, were selected for the gene-gene interaction analysis based on their minor allele frequencies and previous experimental evidence supporting that some of the SNPs have functional effects on NPSR1 expression and/or signaling **(** **Figure 7a** **)**. Allele and genotype distributions for the *NPSR1* SNPs in the combined BAMSE-PARSIFAL material are presented in **Table S1**. An overview of the significant SNP-SNP interactions between *NPSR1* (8 SNPs) and *RORA* (35 SNPs) is presented in **Figure 7a**. We found significant interactions between three non-synonymous SNPs in *NPSR1*, namely rs34705969 (Cys197Phe), rs727162 (Ser241Arg) and rs6972158 (Gln344Arg) and three *RORA* SNPs (rs17191582, rs6494221 and rs12899193) **(** **Figure 7a** **)**. A second hub of strong interaction signals was found between three SNPs located in the *NPSR1* promoter (rs1963499, rs2168890 and rs2530547) and the *RORA* SNP rs746241. An additional interaction signal was detected between the *NPSR1* promoter SNP rs2530547 and *RORA* rs2899662 **(** **Figure 7a** **)**. Interestingly, these *RORA* SNPs did not show any significant *main effects* on asthma, but they had significant effects on asthma susceptibility dependent on *NPSR1.* Furthermore, we identified significant interactions between the *RORA* SNP rs7164773, which showed the most significant SNP *main effect* on childhood asthma, both at 4 and 8 years in BAMSE, and two coding SNPs in the *NPSR1* gene, i.e., rs34705969 (Interaction p = 0.005) and rs6972158 (Interaction p = 0.001). These results suggest that the effects of *RORA* SNPs as risk factors for asthma may differ depending on the *NPSR1* genotype. An example is illustrated in **Figure 7b** for *RORA* rs7164773 and *NPSR1* rs6972158. The CC genotype in *RORA* rs7164773 only confers risk for asthma in carriers of one or two copies of the rare allele G in *NPSR1* rs6972158, encoding for a substitution of glutamine by arginine in exon 9 (OR 2.45, 1.56–3.85, p = 0.00009) but not in the homozygotes for the wild-type *NPSR1* allele A (OR 1.02, 0.66–1.58, p = 0.91). Taken together, we showed that combinations of common susceptibility alleles (in *RORA*) and less common functional polymorphisms (in *NPSR1*) could modify the joint risk effect on asthma susceptibility.

To further interpret the significance of the gene-gene interactions between *RORA* and *NPSR1* in terms of functional effects on signal transduction, we investigated the interactions between *RORA* SNPs and a functional haplotype in *NPSR1* that showed significant association with reduced risk of inflammatory bowel disease in a previous study [47]. The haplotype CAC is a combination of SNPs in *NPSR1* containing the alleles rs2530547**C**/rs324981**A**/rs727162**C** **(** **Figure 7a** **)**; it is found in 9.2% of the population, and corresponds to increased *NPSR1* mRNA expression (−103C) and weaker intracellular signaling (107Asn and Ser241). Although this haplotype was not associated with asthma, significant effects in increasing asthma risk become evident if the haplotype CAC coincided with the rare alleles of the SNPs rs8024629 and rs9302215 in *RORA* **(** **Figure 7c** **)**. The *RORA* SNP rs8024629, only showed significant *main effects* for physician-diagnosed asthma by age 4 years **(** **Table 1** **and File S4)**, but it might also increase the risk for asthma in the combined dataset in the co-occurrence with the functional *NPSR1* haplotype CAC (OR 2.63, 1.30–5.34; p = 0.007). Similar effects were found for the *RORA* SNP rs9302215, with only marginal *main effects* on the risk of physician-diagnosed asthma by age 4 years **(Files S2, S3, S4)**, but that in combination with the functional haplotype of *NPSR1* might significantly increase the risk for asthma in children (OR 2.88; 1.33–6.23, p = 0.007) **(** **Figure 7c** **)**.

## Discussion

Here, we present evidence of functional and gene-gene interaction between RORA and NPSR1. Even though *RORA* was recently identified as an asthma susceptibility gene in a GWAS study [1], its role in asthma pathogenesis is unknown. Using cell models, we found that *RORA* is up-regulated upon activation of the NPS-NPSR1 signaling pathway and that RORA, acting as a suppressor factor, may regulate the activity of the *NPSR1* promoter. Several biological functions, including inflammation and neuronal physiology, are expected to be influenced by the functional link between RORA and NPSR1 [3], [48], [49]. Interestingly, the expression of other genes involved in the circadian clock (e.g. *NPAS2*, *PER1* and *CRY1*) was also up-regulated after stimulation of the NPS/NPSR1 pathway. Furthermore, *Rora* mRNA expression was significantly lower in the lung tissue of *Npsr1* deficient mice when compared to wildtype littermates. The results of the genetic association tests and the gene-gene interaction analysis confirmed that polymorphisms in *RORA* are associated with asthma but their effects may increase or decrease depending on the convergence with polymorphisms in the promoter and the exons of *NPSR1*. Altogether, this study provides evidence of a previously unknown connection between RORA and NPSR1 that involve the regulation of the circadian clock. The gene-gene interaction analysis indicated that particular combinations of common susceptibility alleles (amenable to be detected by GWAS approaches), together with less common functional polymorphisms may modify the effects of a given risk allele on asthma susceptibility.

The genetic association between *RORA* rs11071559 and asthma susceptibility was identified in a GWAS of individuals with European descent [1], and further confirmed in other populations [20], [50]. However, in-depth association analyses of *RORA* SNPs covering the asthma-associated region have not been done. We conducted a genetic association study evaluating the effects of 35 polymorphisms in *RORA* as risk factors for asthma and allergy-related traits in two independent European sample sets. Here, we replicate the effects of *RORA* rs11071559 on asthma-susceptibility and discover additional asthma-susceptibility alleles in the surroundings of this SNP. The fact that the association signals were found in two separate datasets of European children and were further observed in the combined dataset support *RORA* as a susceptibility gene for asthma. Although the associated SNPs were different between BAMSE and PARSIFAL datasets, the most significant signals were driven by SNPs narrowed to a 49 kb region surrounding the SNP rs11071559. In addition, the effects of *RORA* susceptibility alleles might be age-dependent as suggested by stronger associations in BAMSE compared to PARSIFAL. In BAMSE, 77% of asthmatic children were diagnosed before or by the age of four years **(** **Figure 3** **)**. However, the PARSIFAL dataset include children with a physician-diagnosis of asthma ever up to age thirteen, and we may expect a broader spectrum of molecular phenotypes of asthma. This may have influenced our results because phenotype heterogeneity is an important source of confounding in genetic association studies [51], and different pathways are involved in determining early-onset vs. late onset wheezing phenotypes [52]. Further explanations include population sub-structure and power limitations by a smaller sample size in PARSIFAL. Nevertheless, significant allele and genotype associations were detected between the GWAS rs11071559 and physician-diagnosed asthma in PARSIFAL when analyzed as separated dataset. The indications that *RORA* may be related with early-onset asthma were also suggested by findings within the BAMSE cohort showing that the risk effects of some *RORA* SNPs (rs8024629, rs17191596, rs12431690) were stronger for asthma cases diagnosed by age 4 in comparison to cases at age 8 **(File S4)**. Although the underlying mechanisms are unknown, the functional link of RORA with the pathogenesis of early-onset asthma might involve alterations in lung development [12].

The observation that RORA down-regulated the *NPSR1* promoter, together with the up-regulation of clock genes (including *RORA*) upon NPSR1 stimulation, led us to hypothesize that RORA could have biological interactions with *NPSR1*. In cell models, the effect of NPS-stimulation on *RORA* mRNA was modest, but it could be inhibited by a selective NPSR1 receptor antagonist and was dose-dependent **(** **Figure 1a** **and File S1)**. Moreover, this effect was clearly specific for the NPS/NPSR1-pathway since *RORA* mRNA was not up-regulated in NPS-stimulated cells with low NPSR1 expression (data not shown). The fact that NPSR1 stimulation led to up-regulation of *RORA* mRNA and other clock genes is in agreement with the hypothesis suggesting that alterations in the expression of clock genes might lead to circadian disruption and immune dysregulation in chronic inflammatory diseases such as inflammatory bowel disease [53], [54] and rheumatoid arthritis [55], [56]. The increase in the expression of clock genes after NPS stimulation might not be directly related to the development of asthma, but might influence several inflammatory processes related to the disease. There is an increasing number of studies showing that clock genes play an important part in modulating inflammation [45], [57]–[59] and indeed, the hallmarks of asthma, namely chronic inflammation, airway hyper-responsiveness and reversible airway obstruction, exhibit 24 h fluctuations with worsening around 4 AM in comparison to 4 PM [60]. Our results in the lung tissue of wildtype and *Npsr1* deficient mice showed that *Rora* was expressed in a time-dependent fashion and the expression was significantly lower in the lung tissue of *Npsr1* ^−/−^ mice during the early hours of the light period. It is tempting to speculate that the genetic associations of *NPSR1* SNPs with chronic inflammatory diseases might be related to dysregulation of neuroimmune-endocrine pathways controlled by the clock genes.

Since both ***RORA*** and ***NPSR1*** are independently associated with asthma, we evaluated the potential epistasis between polymorphisms in these genes (‘gene-gene interactions’). The analyses were conducted in the combined dataset which allowed us to gain statistical power and increase the accuracy of the estimates. Four *NPSR1* SNPs included in the interaction analysis were selected based on experimental evidence of functional effects on gene expression or downstream signaling [47]. The promoter *NPSR1* SNP rs2530547 (−103) significantly affected luciferase expression in gene reporter assays and ***NPSR1*** mRNA levels in human leukocytes, whereas the non-synonymous ***NPSR1*** SNPs (rs324981/Ile107Asn, rs34705969/Cys197Phe, rs727162/Ser241Arg) affected NPS-induced genome-wide transcriptional profiles (including the clock genes *PER1*, *PER2* and *ARNTL*) and CRE-dependent luciferase activities in transfected human cell lines [47]. The substitution of Cysteine 197 Phenylalanine even exhibited a loss-of-function phenotype in these studies. In addition, we included three additional SNPs in the promoter region of *NPSR1* and a non-synonymous SNP (rs6972158) in *NPSR1* with unknown function but potential to affect transcription factor binding or signal transduction, respectively. The epistasis between *NPSR1* and *RORA* include three exonic ***NPSR1*** SNPs (rs34705969, rs727162, rs6972158) and intronic *RORA* SNPs (Figure 7a). Notably, the association of *RORA* rs7164773 with asthma might depend on the convergence with rare alleles of the *NPSR1* SNPs rs6972158 on chromosome 7 (Figure 7b). Similar results were observed for *RORA* SNP rs8024629 and *RORA* SNP rs9302215 which only increase the risk of asthma in individuals carrying the functional haplotype CAC in *NPSR1* (rs2530547C/rs324981A/rs727162C) (Figure 7c). This haplotype might reduce the expression of downstream target genes, such as *RORA*.

The fact that well-replicated asthma candidate genes (i.e., *IL4*, *GSTP1*, *STAT6*, *ADRB2*, *TNF*, *TGFB1*, etc) [61] are not listed as GWAS hits, and that low-ranked SNPs contribute to predict asthma-related phenotypes in children [52], suggests that gene-gene interactions, gene-environment interactions and epigenetics may be of relevance. It is now more clear that genome-wide association studies may detect the effect of common polymorphisms but cannot detect the effects of less frequent coding mutations and low frequency functional SNPs. In this sense, *RORA* might be the proxy for a pathway, where other genes may be involved but their effects are difficult to detect in the GWAS approach. Epistasis and particular intragenic and intergenic allele combinations are important elements in predicting disease risk and may explain a large proportion of the missing heritability [22]. Under this “combinatory effect” it is required that several variants coincide and affect a biological pathway. The phenotype will be driven by the multiplicative effect of variants conditioning a deleterious outcome. Given the redundancy of the biological systems it is feasible that a large fraction of individuals may carry several susceptibility alleles, but depending on the combinations, the outcome of each allele may increase, decrease or become neutralized. The fact that the associations between *RORA* and asthma are detected in GWAS including a European-American population but not identified in African-American and African-Caribbean samples [20] highlight that the genetic context might determine the effects of any given allele.

In conclusion, genetic polymorphisms in *RORA* are risk factors for childhood asthma and have epistasis with polymorphisms in *NPSR1*. The NPS/NPSR1 pathway has biological interactions with *RORA* and other circadian clock genes which could have effects on the rhythmic occurrence of asthma symptoms. The effect of the susceptibility alleles may depend on particular combinations with one or more genotypes in other genes belonging to similar biological pathways, and could in part explain the complex relationships and contradictions leading to the asthma phenotype.

## Materials and Methods

### Ethics Statement

All the samples were analyzed anonymously. The BAMSE study was approved by the Ethics Committee of Karolinska Institutet, Stockholm, Sweden, and regarding PARSIFAL children, ethical approval for the study, including genetic analyses, was obtained in each country. Written informed consent was obtained from the parents and/or legal guardians.

### Study Population

Genetic association studies were conducted in 2033 children (age 8 years) from the prospective Swedish birth-cohort BAMSE [62], [63], and 1120 children (age 5–13 years) from the European multicenter cross-sectional study PARSIFAL [64]. The selected dataset from the PARSIFAL study includes the two control groups, which have more comparable demographic characteristics to the BAMSE birth cohort than the farmer and Steiner school children. A schematic representation of the phenotypes analyzed in this study and the distribution of cases and controls is presented in **Figure 3**. The effects of individual SNPs on asthma risk were evaluated in BAMSE and PARSIFAL separately as well as in the combined BAMSE-PARSIFAL dataset (n = 3153).

### Phenotype Assessment

In both datasets, information on the clinical outcomes was obtained from questionnaires filled by the parents, except for atopic sensitization which was assessed from plasma samples. In BAMSE, *Asthma ever up to 4 years* was defined as a physician-diagnosed asthma after 3 months of life and up to 4 years of age. *Asthma ever up to 8 years* was defined as a physician-diagnosed asthma after 3 months of life and up to 8 years of age. *Wheezing at 4 years* was considered with at least 1 episode of wheeze during the last 12 months prior to the questionnaire of the 4^th^ year. *Rhinitis at 8 years* was diagnosed if the children had episodes of prolonged sneezing or a runny or blocked nose without a common cold in the last 12 months prior to date of questionnaire 8. *Atopic sensitization* was considered present if a child had levels of allergen-specific IgE >0.35 kU/L to Phadiatop®, a mixture of cat, dog, horse, birch, timothy, mugwort, *Dermatophagoides pteronyssinus*, and *Cladosporium* allergens; (ImmunoCAP™, Thermo Fisher Scientific, Phadia AB, Uppsala, Sweden). In PARSIFAL, *physician-diagnosed asthma* was considered to be present for children reporting ever having been diagnosed with asthma, or with obstructive bronchitis more than once. *Current wheezing* was defined as at least one episode of wheezing during the last 12 months. *Current rhinoconjunctivitis* symptoms were defined as sneezing, runny nose, nasal block-up and itchy eyes in the child during the last 12 months without having a cold at the same time. *Atopic sensitization* was defined as at least one allergen-specific serum IgE test ≥0.35 kU/L against a mixture of common inhalant allergens (Phadiatop®). To define the phenotypes for the combined dataset, corresponding outcomes in BAMSE and PARSIFAL were combined as presented in **Figure 3**.

### SNP Selection, Genotyping and QC

A total of 37 SNPs were genotyped in *RORA*. The SNPs were selected using the tagger algorithm implemented in Haploview [65]. The aim was to analyze the common polymorphisms in the surroundings of the GWAS SNP rs11071559 and refine the genomic interval with the strongest association signals. Primers for multiplex PCR and extension reactions were designed by the SpectroDesigner software (Sequenom GmbH, San Diego, CA, USA) **(Table S2)**. PCR and extension reactions were performed according to manufacturer's standard protocols. The SNP analysis was performed by MALDI-TOF mass spectrometry (matrix-assisted laser desorption/ionisation-time of flight; Sequenom GmbH). Each assay was validated using 24 unrelated Caucasians and 3 CEPH DNA samples as well as 14 trios from the CEU population. Based on successful genotyping 35 SNPs were included for association tests (two *RORA* SNPs, namely rs12591749 and rs1820357 were excluded by failure during genotyping). Success rate for genotyping was 98.8% in BAMSE and 97% in PARSIFAL. For *NPSR1* (MIM: 608595), eight SNPs were genotyped in BAMSE and PARSIFAL as aforementioned. The SNPs were selected according to their minor allele frequencies (MAF≥0.02) and the assumption that polymorphisms in the regulatory and coding gene region are the best candidates to play a causative role in gene function. Previous experimental evidence supports that some of the SNPs have functional effects on NPSR1 expression and/or signaling [47]. The selected polymorphisms included four variants in the predicted promoter and four non-synonymous polymorphisms rs324981 (Ile107Asn); rs34705969 (Phe197Cys); rs727162 (Ser241Arg) and 6972158 (Gln344Arg). Genotype frequencies for all SNPs agreed with the expectations under Hardy-Weinberg equilibrium **(Table S3)**.

### Genetic Association Tests

Linkage disequilibrium among the SNPs was calculated in each dataset and in the combined dataset (n = 3153) using the normalized measure of allelic association (*D’*) implemented in Haploview (http://www.broad.mit.edu/mpg/haploview). Genetic association test were conducted using the SVS v7.6.9 software (Golden Helix Inc.). SNPs were filtered out if call rates (<0.85) and if the Hardy-Weinberg Equilibrium in controls reached (p<0.0001) under χ^2^ test and/or Fisher exact test. Basic allelic effects were analyzed by 2×2 allele-count tests and with the Exact Cochran-Armitage test (assuming near-additive risk). Genotype associations were evaluated under genotypic, additive, dominant, and recessive models. Logistic regression was used to model the effects of one copy (heterozygotes) or two copies (homozygotes) of the mutant allele on asthma status having the wild type genotype as reference category after adjustment by country-of-origin, age category and gender in the independent analysis of PARSIFAL (n = 1120) and in the combined BAMSE-PARSIFAL dataset (n = 3153). When the numbers of rare homozygotes preclude association tests, they were combined with the heterozygotes and the effect of the SNP analyzed in a dominant model (rare homozygote+heterozygote vs. wild-type homozygote). A *P* value ≤0.05 was considered statistically significant. To take the multiple tests performed into account the *P* value of allelic and genotype association analyses are presented after 10.000 permutations. The False Discovery Rate of the Exact Armitage test is reported for the genetic association under additive model (**File S3**).

### Cell Culture and NPS Stimulations

SH-SY5Y human neuroblastoma cells (ATCC, CRL-2266™) were grown in DMEM-GlutaMAX™-I Medium (Invitrogen) supplemented with 10% fetal calf serum, 100 U/ml penicillin and 100 U/ml streptomycin at 37°C in a 5% CO_2_ humidified incubator. Transfections were done with FuGENE reagent (Roche) according to the manufacturers protocols. For making stable clones, SH-SY5Y cells were transfected with a NPSR1-A-GFP plasmid and selected with 500 µg/ml of G418 for 3 weeks. NPSR1-A was amplified from colon cDNA of healthy individuals and fusion products of NPSR1-A with green fluorescent protein (GFP) were made in pcDNA3.1/CT-GFP-TOPO (Invitrogen Life technologies). The construct was verified by sequencing. For RORA regulation studies, the SH-SY5Y cells over-expressing NPSR1 and the SH-SY5Y cells with very low endogenous NPSR1 expression were seeded at 0.5×10^6^ cells/ml to 6-well plates. After 24 h, the clock was entrained with fresh cell media, and the cells were either incubated with or without 100 nM NPS (SFRNGVGTGMKKTSFQRAKS) (MedProbe, Oslo, Norway) for 4 h, 8 h, 12 h, 16 h and 24 h or incubated with a dilution series of NPS (0, 10 nM, 100 nM, 1 µM and 2 µM) for 4 h. SHA 68 (*N*-[(4-fluorophenyl)methyl]tetrahydro-3-oxo-1,1-diphenyl-3*H*-oxazolo[3,4-*a*]pyrazine-7(1*H*)-carboxamide), a selective antagonist of NPSR1 was synthetized as described previously at >95% purity as determined by LC-MS [46], and 3 µM SHA 68 was added 10 min before NPS stimulation. Total cellular RNA was isolated with the RNAeasy Mini Kit (Qiagen, Hilden, Germany). Reverse transcription was performed with TaqMan reverse transcription reagents (Applied Biosystems, Rotkreuz, Switzerland) using random hexamers according to the manufacturer's protocol. The stable human embryonic kidney (HEK293) cell line over-expressing NPSR1-A has been described elsewhere [66]. The cells were cultured in 293 SFM II medium (Gibco/Invitrogen) supplemented with penicillin/streptomycin and constantly cultured under puromycin selection (0.8 µg/ml) (Sigma-Aldrich, St Louise, MO, USA), seeded at 1×10^6^ cells/ml and treated with NPS (1 nM–5 µM) for 6 h.

### Npsr1^−/−^ Mice

Eight weeks old *Npsr1* ^−/−^ male mice [29] or their wildtype male siblings in BALB/c background (N9 with speed congenics) were housed under standard 12-hour light-dark cycle conditions and sacrificed by cervical dislocation at different time points. Lung samples were collected and snap-frozen on dry ice. Total RNA was extracted using Eurozol Reagent (EuroClone, Via Lombordia, Italy) and reverse transcribed to cDNA. *Rora* mRNA expression was measured as previously described [29]. Single-group comparisons were made using the unpaired Student’s t-test. Results are expressed as mean ± SEM. *P*-values <0.05 were considered statistically significant.

### qPCR

The mRNA expression was measured with qRT–PCR using SYBR® Green. The primer sequences are given in **Table S4**. The PCR assay was performed in a total volume of 20 µl, containing cDNA template, 10 µl SYBR® Green PCR Master Mix (Applied Biosystems), 100 nM of each primer and 100 nM of GAPDH or 18S probes, using 7500 Fast Real-Time PCR system (Applied Biosystems) with the following reaction conditions: 50°C for 2 min and 94°C for 10 min; following 45 cycles of 92°C for 14 s and 1 min at 60°C. A dissociation stage was added to the SYBR® Green reactions to confirm primer specificity. Relative quantification and calculation of the range of confidence was performed with the comparative ^ΔΔ^CT method. In the cell line studies, results are shown as relative expression compared with un-stimulated cells and *GAPDH* (Applied Biosystems) was used as an endogenous control. The variation in the Ct levels of *GAPDH* expression was 16.52±0.35 (mean ± SD, n = 48). In the mouse studies, data are presented as relative units indicating a fold-change in *Rora* mRNA expression that was normalized to 18S ribosomal RNA (Applied Biosystems), and was relative to the non-template control calibrator. The variation in the Ct levels of 18S expression was 10.69±0.03 (mean ± SD, n = 49) in the temporal expression experiment **(** **Figure 2A** **)** and 8.62±0.07 (n = 12) in the replication dataset **(** **Figure 2B** **)**.

### Transcription Factor Binding Predictions

Prediction for RORA binding sites in *NPSR1* promoter was conducted using sequence-based methods and phylogenetic approaches. Matrixes were retrieved from Transfac under ID. T01527, T01528, T01529. Promoter of the human *NPSR1* was retrieved between −499 to +200 from the Database of Eukaryotic gene promoters (EPD) (http://epd.vital-it.ch/) and analyzed using MatInspector (http://www.genomatix.de) and Consite (http://asp.ii.uib.no:8090/cgi-bin/CONSITE/consite). Binding propensity on the proximal region of NPSR1 −450 to +50 (NM_207173 and NM_207172) was calculated using PScan (http://159.149.109.9/pscan/).

### Luciferase Assays

To study the effects of RORA expression on the activity of the *NPSR1* promoter, HeLa cells (ATCC CCL2) were growth in DMEM medium supplemented with 10% fetal calf serum and penicillin-streptomycin at a density of 0.5×10^6^/well to 80–90% confluence. Cells were co-transfected with 1 µg of the *NPSR1* promoter coupled to firefly luciferase, 50 ng of pRL-TK Renilla Luciferase reporter vector (Promega, Madison, WI, USA), and 500 ng of a plasmid encoding the cDNA for RORA1 (pCMV6-XL5-RORA1, Origene Inc.). The control of this experiment were the HeLa cells co-transfected with 500 ng of the vector pCMV6-XL5 without RORA1 (Cat. PCMV6XL5, OriGene Inc). Transfections were done using the XtremeGene 9 reagent in a 3∶1 ratio (Promega). Total cell lysates were prepared 24 h post transfection using passive lysis. Luciferase activity was calculated using the Dual-Luciferase Reporter Assay System (Promega) according to manufacturer's instructions, and a Microplate Reader Infinite 200 (Tecan, Männedorf, CH). Ten biological replicates were included for each plasmid (RORA1 vs. vector without RORA1). Firefly Luciferase activity was expressed in luciferase units relative to the transfection with the empty vector, after normalization for transfection efficiency based on values obtained for Renilla Luciferase. To obtain a relative estimation of the activity of the *NPSR1* promoter, each experiment was done in parallel with co-transfections of HeLa cells with plasmids encoding the transcription factor *p53* (*p53wt*), the *p53* responsive element coupled to firefly luciferase (RE-luc) or both (*p53wt*+*p53RE-luc*). Transfection of the *p53RE-luc* construct resulted in 4.7-fold more luciferase activity when compared with the *NPSR1* promoter activity (data not shown).

### Gene-gene Interactions

Eight *NPSR1* SNPs were selected to conduct gene-gene interaction test with 35 *RORA* SNPs in the combined dataset. The *NPSR1* SNPs included the promoter polymorphism rs2530547 which affect *NPSR1* gene expression and is located at 307 bp of a RORA binding site, as well as non-synonymous polymorphisms rs324981 (Asn107Ile), rs727162 (Ser241Arg) and rs34705969 (Cys197Phe) with a loss-of-function phenotype (197Phe), which had effects on NPSR1 downstream signaling [47]. In addition, the non-synonymous polymorphism rs6972158 (Gln344Arg) was selected for analyses. The interaction analysis was performed in the pooled BAMSE-PARSIFAL dataset, including 3153 children. A multiple logistic regression model was used to test for gene–gene interaction between *RORA* and *NPSR1* SNPs by adding an interaction term between the genotypes of interest, using STATA (Statistical Software, Version 8.0, Collage Station, TX, USA). A genetic model-free coding with indicator (dummy) variables for common homozygous (coded 0), heterozygous (coded 1) and rare homozygous (coded 2) was used as a first approach to investigate the pattern of interaction between *RORA* and *NPSR1* genotypes. For analyses of SNPs with relatively low minor allele frequency (thus few rare homozygotes) rare homozygotes were combined with the heterozygotes and a dominant genetic model was used. *P*-values for departure from a multiplicative interaction model on the OR scale were obtained by likelihood-ratio tests between the models with and without interaction term. The regression models were adjusted for country-of-origin and age. For the gene-gene interaction analysis a *P* value ≤0.05 was considered statistically significant. However, considering the number of comparisons performed (n = 280), we focused on interactions with a *P* value ≤0.01.

## Supporting Information

Table S1 Allele and genotype distributions of *NPSR1* SNPs in cases and controls (combined dataset).
(PDF)

Table S2 Primer sequences for the genotyping of *RORA* SNPs.
(XLSX)

Table S3 Descriptive information and HWE on the 35 *RORA* SNPs analyzed in this study.
(XLSX)

Table S4 Primers used for qPCR analysis in the human cell lines and mice experiments.
(DOCX)

File S1 Stimulation of human SH-SY5Y neuroblastoma cell line over-expressing NPSR1 with increasing doses of the ligand NPS.
(PDF)

File S2 Allelic association tests for 35 *RORA* SNPs in BAMSE, PARSIFAL and the combined dataset.
(XLSX)

File S3 Genotype association tests for 35 *RORA* SNPs in BAMSE, PARSIFAL and the combined dataset under *additive*, *dominant, genotypic* and *recessive* models.
(XLSX)

File S4 Genotype distribution of the *RORA* SNPs significantly associated with physician-diagnosed asthma in BAMSE, PARSIFAL and the combined dataset.
(XLSX)
