# Supplementary material for: Interaction between Retinoid Acid Receptor-Related Orphan Receptor Alpha (RORA) and Neuropeptide S Receptor 1 (NPSR1) in Asthma
Source: PLoS One. 2013 Apr 2;8(4):e60111. doi: 10.1371/journal.pone.0060111 (PMC3615072; doi:10.1371/journal.pone.0060111)
Supplement: Table S1 — Allele and genotype distributions of NPSR1 SNPs in cases and controls (combined dataset). (PDF) [file pone.0060111.s001.pdf]

Supplementary table S1. Allele and genotype distributions of *NPSR1* SNPs in cases and controls (combined dataset)

				Controls (n = 2631)	Cases (n = 426)				Controls (n = 2631)	Cases (n = 426)			
**db SNP**	**Coordinate**	**SNP**	**Location**	**MAF**	**MAF**	**OR (95%CI)**	**Fisher's Exact P**	**Genotype**	**n (%)**	**n (%)**	**adj P value***	**aOR (95%CI)**	**adj P value****
rs1963499	34663985	G/A	Promoter	0.098	0.10	1.08 (0.85-1.37)	0.53	GG	2057 (81.1)	329 (79.9)	0.80	1.0	-
								GA	457 (18.0)	79 (19.2)		1.06 (0.81-1.39)	0.63
								AA	21 (0.8)	4 (1.0)		1.19 (0.40-3.51)	0.74
								GG vs GA + AA	478 (18.9)	83 (20.1)	0.53	1.07 (0.82-1.39	0.60
rs2168890	34663997	C/T	Promoter	0.11	0.11	0.99 (0.78-1.25)	1	CC	1891 (77.5)	305 (78.0)	0.83	1.0	-
								CT	512 (21.0)	79 (20.2)		0.95 (0.73-1.24)	0.72
								TT	36 (1.5)	7 (1.8)		1.24 (0.54-2.83)	0.59
								CC vs CT + TT	548 (22.5)	86 (22.0)	0.83	0.97 (0.75-1.25)	0.82
rs2530547	34664447	C/T	Promoter	0.35	0.37	1.12 (0.96-1.30)	0.14	CC	1071 (42.9)	161 (39.2)	0.34	1.0	-
								CT	1091 (43.7)	188 (45.7)		1.15 (0.92-1.44)	0.21
								TT	336 (13.5)	62 (15.1)		1.24 (0.90-1.71)	0.17
								CC vs CT + TT	1427 (57.1)	250 (60.8)	0.15	1.17 (0.95-1.45)	0.13
rs887020	34664507	G/A	Promoter	0.42	0.47	1.21 (1.03-1.42)	0.015	GG	778 (34.4)	105 (28.5)	0.05	1.0	-
								GA	1056 (46.7)	180 (48.9)		1.28 (0.99-1.66)	0.058
								AA	426 (18.8)	83 (22.6)		1.46 (1.07-2.00)	**0.017**
								GG vs GA + AA	1482 (65.6)	263 (71.5)	0.026	1.33 (1.04-1.70)	**0.019**
rs324981	34784638	A/T	Exon 3	0.46	0.47	1.01 (0.87-1.17)	0.84	AA	725 (28.3)	119 (29.4)	0.51	1.0	-
								AT	1276 (49.9)	190 (46.9)		0.90 (0.70-1.15)	0.42
								TT	558 (21.8)	96 (23.7)		1.04 (0.77-1.39)	0.78
								AA vs AT + TT	1834 (71.7)	286 (70.6)	0.66	0.94 (0.75-1.19)	0.64
rs34705969	34833649	G/T	Exon 5	0.026	0.025	0.94 (0.59-1.50)	0.90	GG	2421 (94.7)	392 (94.9)	0.92	1.0	-
								GT	135 (5.3)	21 (5.1)		0.94 (0.58-1.51)	0.80
								TT	1 (0.0)	0 (0.0)		-	
								GG vs GT + TT	136 (5.3)	21 (5.1)	0.84	0.93 (0.58-1.49)	0.77
rs727162	34840563	C/G	Exon 6	0.21	0.20	0.95 (0.79-1.14)	0.61	CC	1548 (61.5)	262 (64.4)	0.17	1.0	-
								CG	854 (33.9)	121 (29.7)		0.83 (0.66-1.05)	0.12
								GG	117 (4.6)	24 (5.9)		1.18 (0.74-1.87)	0.46
								CC vs CG + GG	971 (38.5)	145 (35.6)	0.26	0.87 (0.70-1.09)	0.24
rs6972158	34855707	A/G	Exon 9a	0.34	0.33	0.96 (0.82-1.12)	0.66	AA	1103 (43.2)	187 (45.7)	0.43	1.0	-
								AG	1162 (45.5)	172 (42.1)		0.87 (0.70-1.09)	0.24
								GG	291 (11.4)	50 (12.2)		1.03 (0.73-1.45)	0.84
								AA vs AG + GG	1453 (56.8)	222 (54.3)	0.33	0.90 (0.73-1.11)	0.35

MAF: Minor Allele frequency

adj P value*= after 10000 sampled tables

adj P value** = country-of-origin and age group
