# Supplementary material for: Interaction between Retinoid Acid Receptor-Related Orphan Receptor Alpha (RORA) and Neuropeptide S Receptor 1 (NPSR1) in Asthma
Source: PLoS One. 2013 Apr 2;8(4):e60111. doi: 10.1371/journal.pone.0060111 (PMC3615072; doi:10.1371/journal.pone.0060111)
Supplement: Table S4 — Primers used for qPCR analysis in the human cell lines and mice experiments. (DOCX) [file pone.0060111.s004.docx]

**Supplementary table S4**. Primers used for qPCR analysis in the human cell lines and mice experiments

| Human cell lines | |  |  |  |
| --- | --- | --- | --- | --- |
| Gene | Accession | 5' Primer | 3' Primer | Length |
| *ARNTL* | NM_001178 | CTGGAGAAGGTGGCCCAAAGAG | CCACTGGAAGGAATGTCTGGAGTC | 250 |
| *CLOCK* | NM_004898 | TTCTGCCTCTTCTCGGAGTTCAAG | CCTGGGTGGAGTGCTCGTATC | 103 |
| *NPAS2* | NM_002518 | CTTCCCTGCCTCCCAACCATC | GGTCCCTGGCTGTTGTGAGTAG | 151 |
| *PER1* | NM_002616 | CTCCAATCAGGACGCACTTTC | GCTGCCAAAGTATTTGCTTGTG | 211 |
| *CRY1* | NM_004075 | TCTGGCATCAGTACCTTCTAATCC | CTGTGTGTCCTCTTCCTGACTAG | 226 |
| *RORA* | NM_134262.2 | CCAGCCCCGACGTCTTCAAAT | GCCATGAGCGATCTGCTGACA | 150 |
| *NR1D1* | NM_021724 | CTTGGCTGCCCAGCGTCATAAC | CCAGATCTCCTGCACCGTTCG | 274 |
| *DBP* | NM_001352 | CTTAAGCCCCAGCCAATCATGAAG | CCGCCCGCACCGATATCTG | 160 |
|  |  |  |  |  |
| *NPSR1 ^-/-^ mice model* | |  |  |  |
| mm*Rora* | [NM_013646.1](http://www.ncbi.nlm.nih.gov/entrez/viewer.fcgi?db=nucleotide&id=7305438) | TGCGAGCTCCAGCCGAGGTA | GCCCTTGCAGCCTTCACACGTA | 136 |
