# Supplementary material for: Interaction between Retinoid Acid Receptor-Related Orphan Receptor Alpha (RORA) and Neuropeptide S Receptor 1 (NPSR1) in Asthma
Source: PLoS One. 2013 Apr 2;8(4):e60111. doi: 10.1371/journal.pone.0060111 (PMC3615072; doi:10.1371/journal.pone.0060111)
Supplement: File S1 — Stimulation of human SH-SY5Y neuroblastoma cell line over-expressing NPSR1 with increasing doses of the ligand NPS. (PDF) [file pone.0060111.s005.pdf]

Supplementary figure S1.

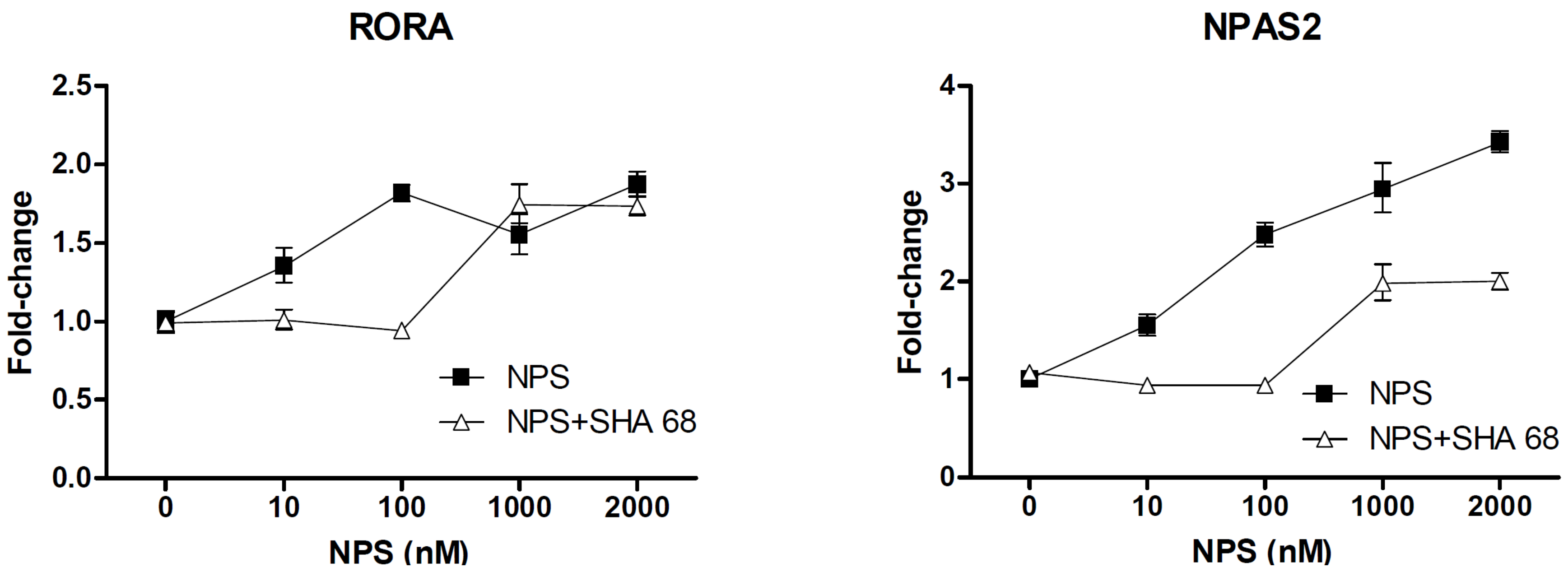

Stimulation of human SH-SY5Y neuroblastoma cell line over-expressing NPSR1 with increasing doses of the ligand NPS. Real-time PCR analysis for the mRNA expression of *RORA* and *NPAS2* with and without the NPSR1 antagonist SHA 68.
